# Understanding the Performance of a Novel Direct Compression Excipient Comprising Roller Compacted Chitin

**DOI:** 10.3390/md18020115

**Published:** 2020-02-17

**Authors:** Deeb Abu Fara, Linda Al-Hmoud, Iyad Rashid, Babur Z. Chowdhry, Adnan Badwan

**Affiliations:** 1 Chemical Engineering Department, School of Engineering, University of Jordan, Amman 11942, Jordan; l.alhmoud@ju.edu.jo; 2 Research and Innovation Centre, The Jordanian Pharmaceutical Manufacturing Company (JPM), P.O. Box 94, Naor 11710, Jordan; irashid@jpm.com.jo (I.R.); adnanbadwan@gmail.com (A.B.); 3 School of Science, Faculty of Engineering & Science, University of Greenwich, Medway Campus, Chatham Maritime, Kent ME4 4TB, UK; b.z.chowdhry@greenwich.ac.uk

**Keywords:** chitin, roller compaction, ball milling, direct compression, compression work, crushing strength, Hausner ratio, Kawakita analysis, bulk density, dissolution

## Abstract

Chitin has been investigated in the context of finding new excipients suitable for direct compression, when subjected to roller compaction. Ball milling was concurrently carried out to compare effects from different energy or stress-inducing techniques. Samples of chitin powders (raw, processed, dried and humidified) were compared for variations in morphology, X-ray diffraction patterns, densities, FT-IR, flowability, compressibility and compactibility. Results confirmed the suitability of roller compaction to convert the fluffy powder of raw chitin to a bulky material with improved flow. X-ray powder diffraction studies showed that, in contrast to the high decrease in crystallinity upon ball milling, roller compaction manifested a slight deformation in the crystal lattice. Moreover, the new excipient showed high resistance to compression, due to the high compactibility of the granules formed. This was correlated to the significant extent of plastic deformation compared to the raw and ball milled forms of chitin. On the other hand, drying and humidification of raw and processed materials presented no added value to the compressibility and compactibility of the directly compressed excipient. Finally, compacted chitin showed direct compression similarity with microcrystalline cellulose when formulated with metronidazole (200 mg) without affecting the immediate drug release action of the drug.

## 1. Introduction

Pharmaceutical excipients for direct compression (DC) applications are mostly favored in relation to saving time, cost and labour for solid dosage form preparations and tableting [1,2]. The foregoing advantages are due to their ability to provide the three main requirements associated with excipients for DC processing, i.e., compressibility, compactibility, and flowability [3,4]. Many DC excipients are manufactured from natural sources (e.g., cellulose and starch), from existing excipients of synthetic origin or from binary mixtures of non-DC excipients [5,6]. The necessity for structural modification and industrial manufacture is attributed to the detrimental physical properties of most pharmaceutical excipients before being processed. These properties include poor compactibility, compressibility, and flowability. 

Industrially, different processes have been used in the scale-up production of DC excipients. Spray-drying and spray-granulation represent the most two common processes in DC excipient production [7,8,9]. However, apart from the high cost and investment of time, these techniques commonly impose complexity in terms of operational procedures, as well as process control [10]. Moreover, prior to spray drying, most excipients are subjected to physical and chemical treatment in order to provide specific functionalities for in vivo drug delivery purposes [11,12]. Such pre-treatment steps add to the complexity of product manufacture.

Dry granulation represents a preferred industrial alternative in order to minimize time and cost for a myriad of pharmaceutical applications. This is due to the fact that neither liquids nor heat is involved in the dry processing of powders. Arguably, the most promising dry granulation technique, to date, is roller compaction, since it has proved to be effective in replacing powders that are conventionally processed using wet granulation [13,14]. However, most applications of roller compactors are confined to the improvement of powder flow of pharmaceutical preparations comprising mixtures of API(s) and excipient(s) [15]. Nevertheless, there have been attempts to employ roller compaction technology for the conversion of poorly compressible/compactable starch and α-lactose monohydrate into DC excipients [16,17]. In this regard, specific intensive compaction pressures were able to produce DC excipients via the mechanism of gelatinization and reduction in crystallinity for starch and α-lactose monohydrate, respectively. 

Recently, there has been a significant interest in the development of chitin for pharmaceutical use, especially in direct compression processing. The basic asset of chitin that renders such a development to be advantageous lies in its ability to provide vital multi-functionalities in tablet processing. In this regard, chitin showed good tabletability, fast disintegration properties, in addition to improved flowability, compressibility, and compactibility when processed with other common excipients, such as calcium carbonate and magnesium silicate [18,19,20,21,22,23]. Despite the foregoing comments, chitin lacks essential manufacturing requirements for the processing of DC excipients. In this regard, the low bulk density and poor powder flowability represent the two major inherent shortcomings of chitin. Nevertheless, numerous attempts have been made to convert chitin into a pharmaceutical DC excipient. Most of these attempts have adopted co-processing techniques, whereby another excipient has been involved in, e.g., wet granulation methodologies for product manufacturing [20,23]. However, the manufacturing procedures and processing time and cost of such methodologies are, relatively, complex. This necessitates searching for new technical alternatives for the processing of chitin in order for it to be used as an excipient with DC functionality. 

The research reported herein attempts to extend the usefulness and opportunities that roller compaction may provide in obtaining a new DC excipient using chitin. Because roller compaction is a pressure inducing technique, it was concurrently compared with ball milling in order to further support an understanding of the performance of modified chitin, as an excipient, when subjected to pressure.

## 2. Results 

### 2.1. SEM

**Figure 1:**
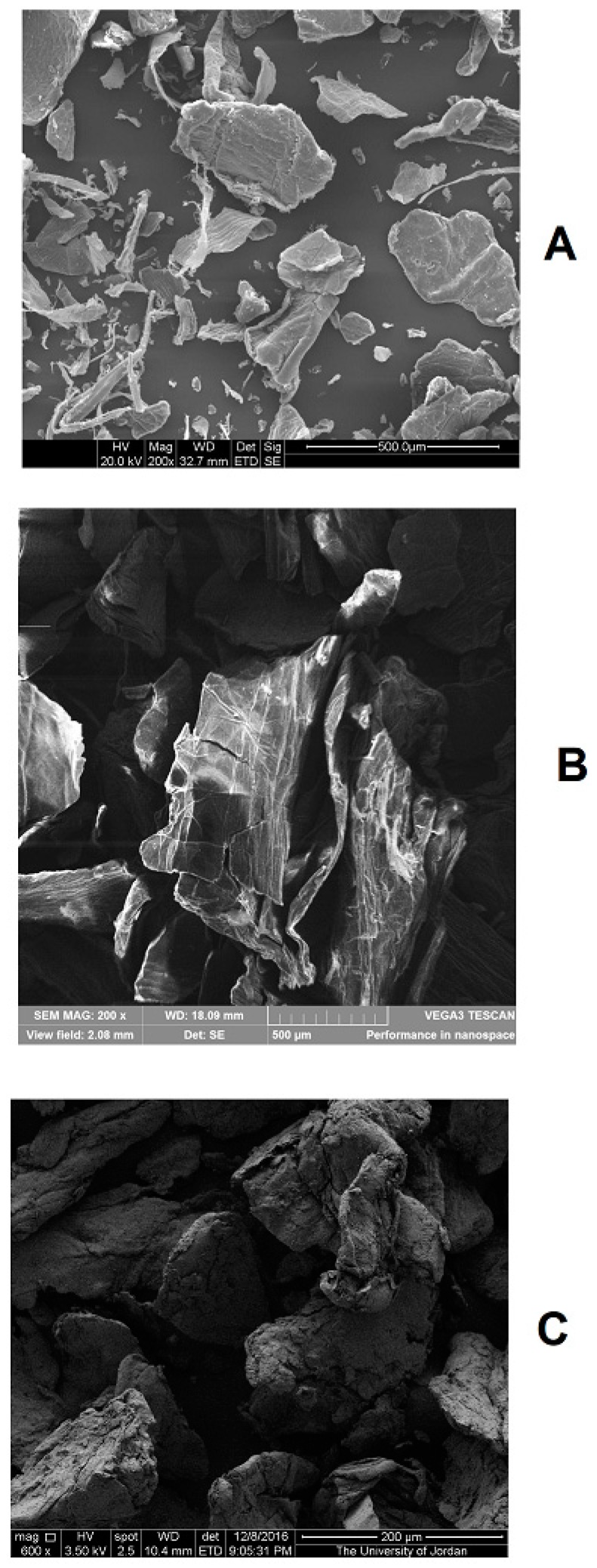
SEM images of raw chitin (**A**), ball milled chitin (**B**), and compacted chitin (**C**).

SEM of raw, ball milled, and compacted chitin particles display the morphology presented in Figure 1. Originally, the raw chitin particles are thin, and most of their surfaces are flat with some degree of folding. The shape did not change dramatically upon ball milling; however, the surface of the particles became more flattened with some degree of surface damage and tearing. In contrast, compacted chitin particles were thick and displayed a high degree of surface irregularities.

### 2.2. XRPD Analysis

**Figure 2:**
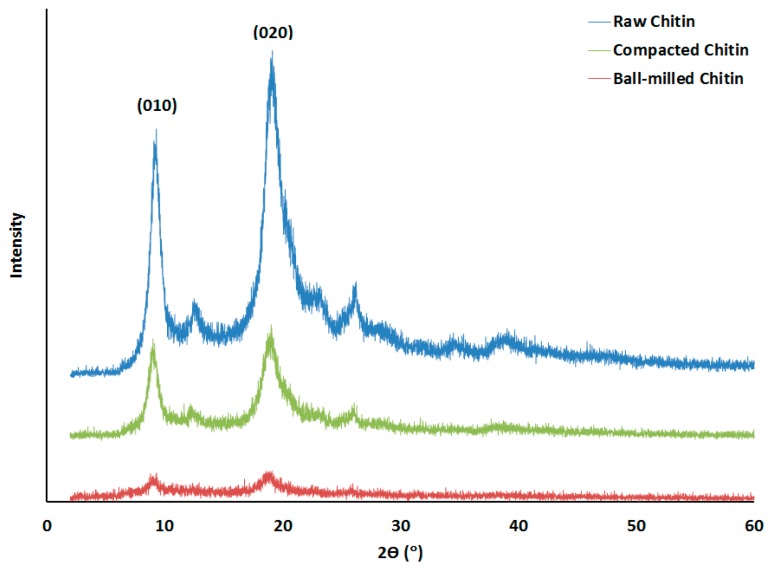
XRPD spectra of raw chitin (blue), and that subjected to roller compaction (green) and ball milling (red).

The XRPD spectra of raw chitin, and that subjected to roller compaction and ball milling for 36 h, is presented in Figure 2. Initially, the pattern for the raw chitin shows two main sharp peaks indicative of α-chitin at 2θ = 9° and 19°, whereby the intensity of the peak at 19° is higher than that at 9° [24]. It is obvious that ball milling decreased the intensities of these much more than roller compaction indicating the sever action of the ball milling process. When the area under the diffraction peaks are considered for the two planes (010) and (020), the summation of the areas are 1984, 787, and 174 for raw, compacted, and ball milled chitin powders, respectively (Table 1). 

### 2.3. FTIR Spectrophotometry 

**Figure 3:**
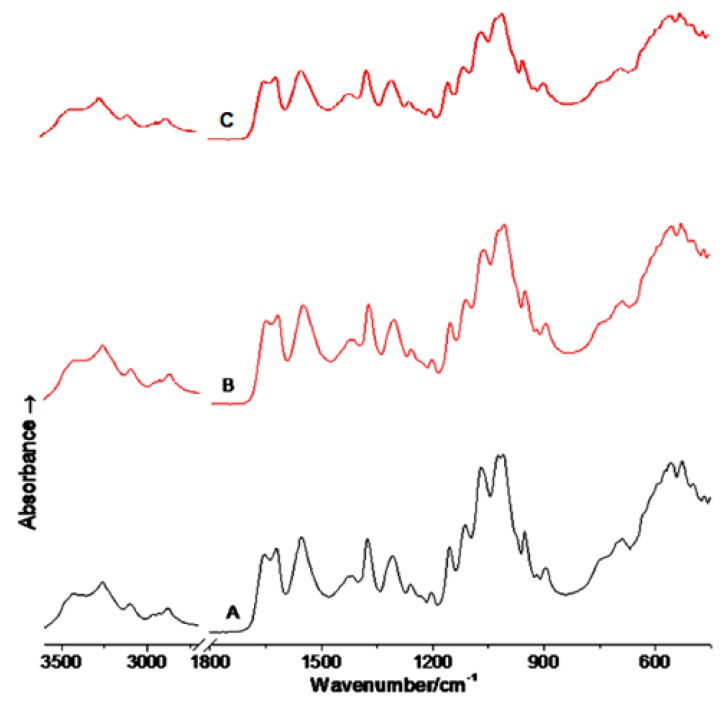
IR spectra of raw chitin (**A**), compacted chitin (stage 5) (**B**), and ball milled chitin (36 h) (**C**).

FTIR spectra of raw, ball milled, and compacted chitin samples are presented Figure 3A–C, respectively. The main characteristic bands of chitin (Figure 3A) were detected at 1620 and 1660 cm^−1^ for amide I and at 1560 cm^−1^ for amide II regions. These bands did not change when chitin was subjected to roller compaction (Figure 3B) and ball milling (Figure 3C). However, there was broadening and a decrease in band intensities for the identity bands of ball milled chitin. 

### 2.4. Bulk, Tapped Density and True Density

The bulk and tapped densities of the light, fibrous raw chitin material increased with ball milling and compaction, whereby the later technique produced the densest powder (Table 2). Bulk and tapped densities were found to be affected by the number of water molecules within the chitin powder. In this regard, when the powder was subjected to humidification, under 93% RH for 30 days, the measured bulk density underwent a decrease for all three samples of chitin. In contrast, drying of the samples at 95 °C for four days caused an increase in bulk and tapped densities for all types of powders; raw-unprocessed, ball milled, and compacted.

The true density of chitin raw material underwent a decrease by 8% when the raw material was subjected to ball milling; the results are also illustrated in Table 2. In the same regard, roller compaction did not change the true density of chitin. Nevertheless, a further decrease in true density of the raw material was recorded when it was subjected to humidity conditions. However, true density values were the highest when the raw and processed materials were dried at 95 °C for four days. 

### 2.5. Particle Size Distribution 

Results of the particle size analysis of raw, ball milled, and compacted chitin are illustrated in Table 3. Ball milling was able to reduce the particle size of the raw material of chitin (d_0.5_ = 613 μm) to a value of d_0.5_ = 384 μm, whereas, roller compaction increased the particle size to a value of d_0.5_ = 877 μm. These values were the actual particle sizes resulting from roller compaction and ball milling. As such, the values are larger than the particle size distribution for common DC excipients, e.g., lactose DC and Avicel^®^ 200 [25,26]. Therefore, all powders subjected to investigation, including processed and unprocessed chitin were passed over a mesh size 250 μm and collected on a 90 μm mesh. The new particle size distribution after sieving is presented in Table 3.

### 2.6. Hauser Ratio

The Hausner ratios of all types of chitin powders (unprocessed, balled milled, and roller compacted), are presented in Table 4. Chitin, and to the same extent, ball milled chitin displayed poor flowability (HR > 1.45). However, roller compaction improved the powder flow to ‘fair’ criteria (HR; 1.19–1.25). Such an improvement was further noticed when chitin, as raw material, was subjected to humidity conditions. In this regard, a ‘passable’ flow criteria was recorded (HR: 1.26–1.34). In contrast, drying resulted in powders with poor flow property. A similar observation in flow behavior when the powder was humidified and dried was noticed for ball milled chitin, whereby the dried powders presented poor flow. However, the observation was the opposite for roller compacted chitin. In this regard, dried powders of this type showed the best improvement in powder flow where an ‘excellent’ flow criteria were recorded (HR < 1.11). In the same regard, a poor powder flow was recorded when the compacted powder was subjected to humidity conditions. 

### 2.7. Water Content 

Results of the Karl Fischer water content for the chitin samples are presented in Table 5. The test clearly shows that humidification doubled the amount of water content from its initial value at room temperature for raw and processed chitin. In contrast, water content was reduced when the samples underwent drying. The decrease was more enhanced for processed chitin than for the raw material. 

### 2.8. Specific Surface Area

Specific surface area measurements give an indication in the difference between the two particle deformation techniques, ball milling and compaction. Results of these measurements are presented in Table 6 for the raw, ball milled, and compacted chitin powders. As expected, raw chitin showed a high specific surface area which underwent an increase or a decrease when the powder was subjected to ball milling or roller compaction, respectively.

### 2.9. Tablet Crushing Force

**Figure 4:**
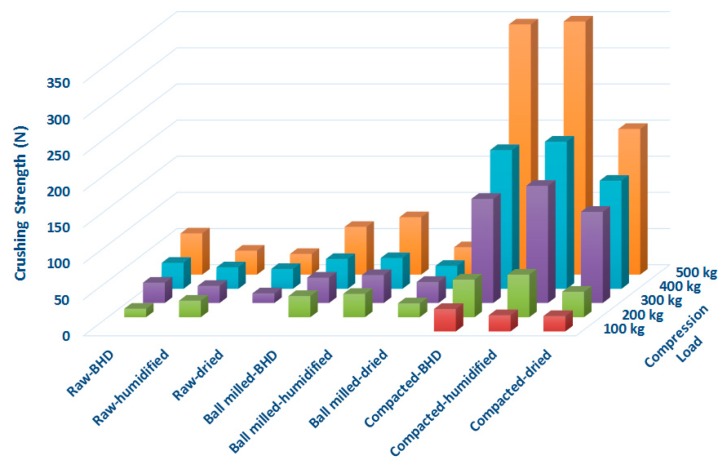
Crushing strength of tablet made of raw (unprocessed), ball milled, and compacted chitin, before and after humidification or drying [BHD: before humidification or drying].

Results for the tablet crushing force when the different powders were compressed into 6 mm diameter tablets (75 ± 1 mg each) using the GTP at compression loads of 100 to 500 kg (34.67–173.35 MPa pressure), are presented in Figure 4. At a compression force of 100 kg (34.67 Mpa), neither the raw nor the ball milled chitin powders (humidified and dried) were able to be compressed into tablets. The aforementioned materials started to form proper tablets at 200 kg of compression load (69.34 Mpa). When the foregoing was increased, the crushing force, ultimately, underwent an increase. Within the same range of compression load, i.e., 100 to 500 kg (34.67–173.35 Mpa pressure), the crushing force of tablets made using roller compacted powder was significantly higher than tablets made of raw and ball milled chitin. These results indicate the high compactibility of chitin when subjected to roller compaction. On the other hand, the data in Figure 4 further indicates that all types of humidified powders (raw-unprocessed and processed) produce tablets with higher crushing force than raw and dried materials.

### 2.10. Kawakita Compression Analysis

**Figure 5:**
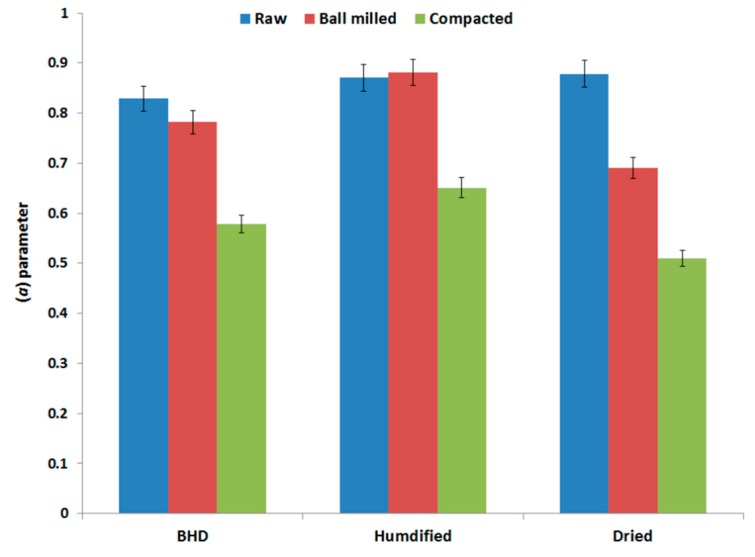
Kawakita parameter (*a*) of raw (unprocessed), ball milled, and compacted chitin, before and after humidification or drying [BHD: before humidification or drying].

**Figure 6:**
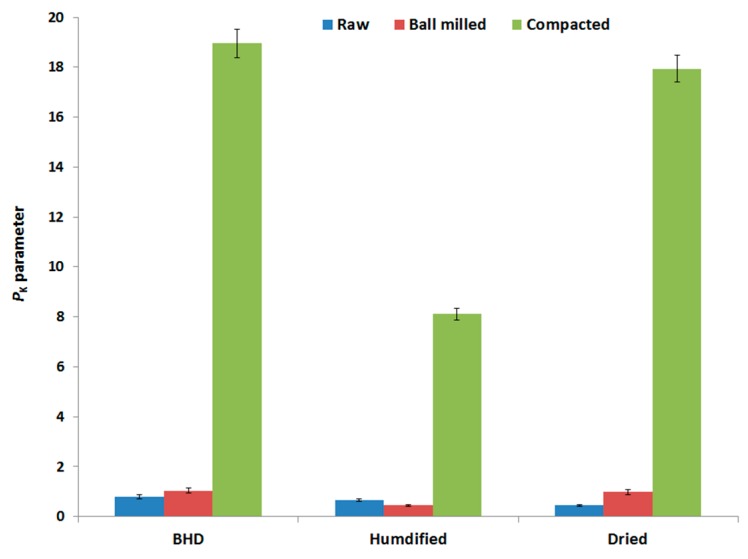
Kawakita parameter (*p_k_*) of raw (unprocessed), ball milled, and compacted chitin, before and after humidification or drying [BHD: before humidification or drying].

**Figure 7:**
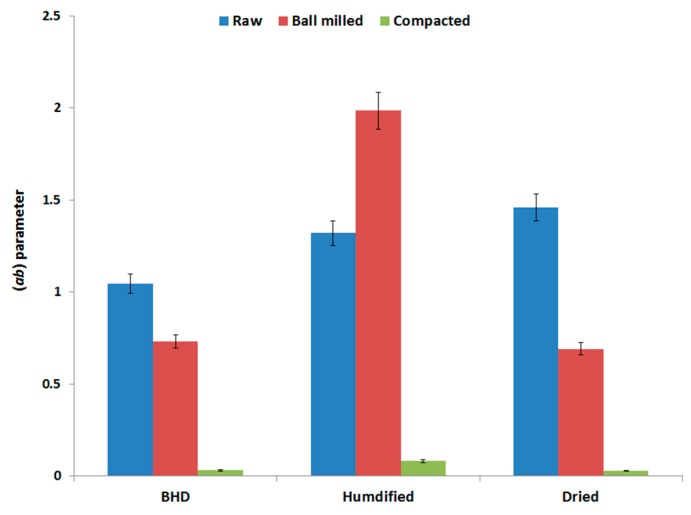
Kawakita parameter (*ab*) of raw (unprocessed), ball milled, and compacted chitin, before and after humidification or drying [BHD: before humidification or drying].

The three main parameters (*a*, *P_k_* and *ab*) obtained via Kawakita analysis (explained in Section 4.2.5 of the method section) were analyzed in an attempt to interpret the compression behavior of the three samples of chitin. The values of each parameter for each powder type (raw and processed) under the two set conditions (humidified and dried) are presented in Figure 5, Figure 6 and Figure 7.

The maximum volume reduction that can be attained (*a*) is presented in Figure 5 and illustrates that compacted chitin underwent the lowest volume reduction when a compression force was applied compared to raw and ball milled chitin. For the latter two materials, volume reduction of raw-unprocessed chitin was the highest followed by ball milled chitin. Furthermore, the two types of processed chitin—compared to their initial status (pre-drying and pre-humidification)—underwent either an increase in volume reduction when they were subjected to humidification, or a decrease upon drying. 

*P_k_*, which represents the pressure needed to reduce (*a*) into half its initial value, is the most important Kawakita parameter to be tested. This is due to the fact that it represents how hard the granules are, and therefore, their ability to be used in direct compression applications [27]. The data in Figure 6 shows that the *P_k_* values of compacted raw chitin powder were the highest amongst all three types of samples. Ball milling causes a slight increase in the *P_k_* value compared to the raw material. However, such an increase is not comparable to roll compacted powder. It is worth noting that although humidification improved the compressibility of all the powders, *P*_K_ values were dramatically reduced even for roller compacted chitin. With regard to dried powders, drying caused a small decrease in *P_K_* values for all the powders when compared with non-dried samples. 

The last Kawakita parameter that was used in this work to describe the compression behavior is *ab*. This parameter gives an indication of the degree of rearrangement of powder particles [28,29], Figure 7. Compared to the raw material, processing of chitin either by ball milling or by roller compaction reduced the extent of particle rearrangement (*ab*) upon compression. In the same regard, the value of *ab* was the lowest for the roller compacted powder. The data in Figure 7 also indicates that humidification increased the extent of particle rearrangement, especially for ball milled chitin, whereas, the values for *ab* for dried powders (ball milled and compacted) were almost similar to the values of the powders in the pre-dried state.

### 2.11. Heckel Compression Analysis

**Figure 8:**
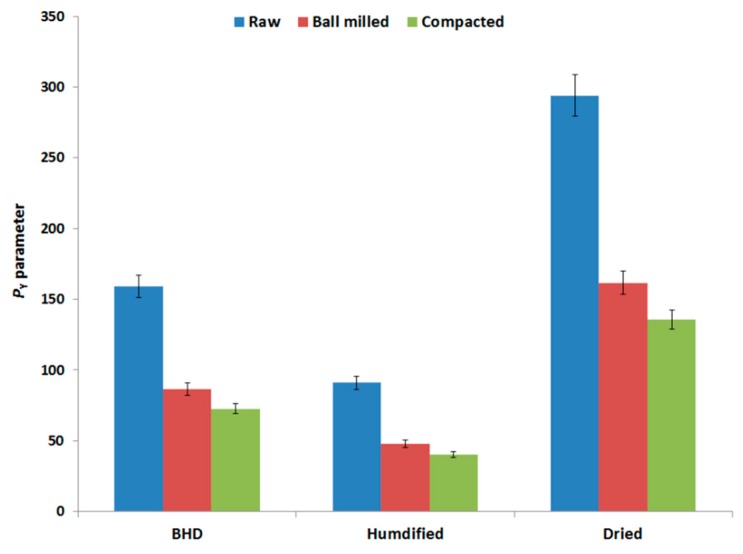
Heckel parameter (*P_Y_*) of raw (unprocessed), ball milled, and compacted chitin, before and after humidification or drying [BHD: before humidification or drying].

Compression analysis was further examined using the empirical Heckel model of compression analysis. In this model, the yield pressure, or *P_Y_*, represents a critical parameter which reflects the type and extent of deformation, i.e., plastic/elastic or brittle-fracture. The data in Figure 8 illustrates the yield pressure values for all the powders tested. Results show that when the two stress-inducing techniques are compared with each other and with the raw material, the *P_Y_* ranking followed the order: Raw chitin > ball milled chitin > roller compacted chitin. Humidification of each type of powder (raw and processed) further reduced the value of *P_Y_*. Moreover, *P_Y_* values of the three types of chitin (raw-unprocessed, ball milled, and compacted) underwent an increase upon drying.

### 2.12. Work of Compression

**Figure 9:**
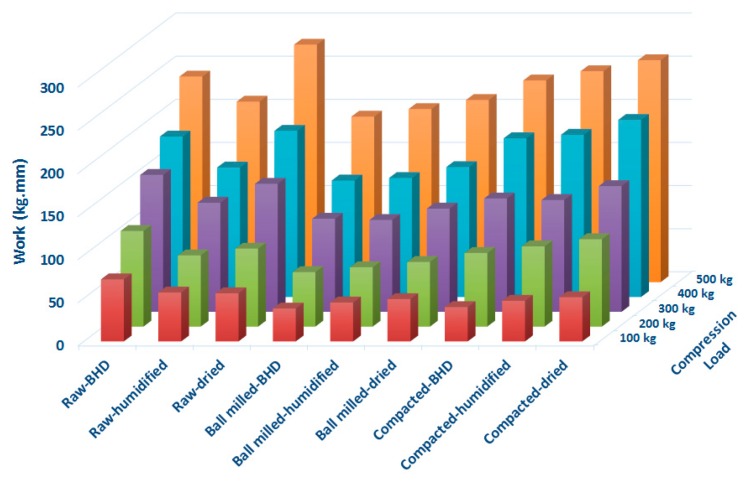
Compression work for raw (unprocessed), ball milled, and compacted chitin, before and after humidification or drying [BHD: before humidification or drying].

When powders of different types were compressed using the GTP, the instrument displays the force displacement curve during the descending/compression and decompression of the powders. The work of compression (*W_c_*), which is represented by the area under the compression curves of the F-D profiles, is shown in Figure 9. *W_c_* values were calculated at each compression force used. Results indicate that at all loads, *W_c_* of raw-unprocessed chitin > *W_c_* of roller compacted chitin > *W_c_* of ball milled chitin. Moreover, above 300 kg of compression load (i.e., at 400 and 500 kg), drying of raw and processed materials rendered *W_c_* higher than both humidified and un-dried powders.

### 2.13. Characterization of Metronidazole Tablets Comprising Drug/Compacted-Chitin Matrix

**Figure 10:**
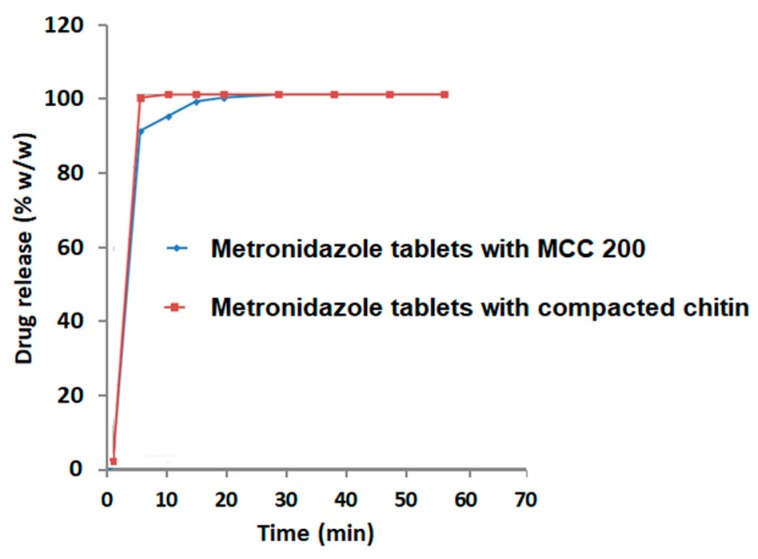
Dissolution profiles for metronidazole (200 mg) tablets comprising compacted chitin or MCC 200^®^ excipients.

Tablet properties and dissolution are illustrated in Table 7 and Figure 10. These results demonstrate that tablets comprising chitin are harder in terms of crushing force and faster in disintegration time than tablets made of MCC PH 200^®^. Moreover, full drug release of metronidazole/chitin matrix was achieved within 5 min of dissolution time. This was faster than tablets comprising metronidazole/MCC matrix, which resulted in full drug release within 20 min of dissolution time. 

## 3. Discussion

The research presented herein is mainly focused on the assessment of the use of roller compaction technique for the development of DC pharmaceutical excipients from raw alpha chitin. Ball milling, as another stress-inducing technique, was tested for comparison and evaluation of changes in the physical properties and compression behavior of chitin powder. The main challenge in this work is to obtain workable chitin with suitable DC properties to facilitate compaction and compression in the industrial set up. FTIR test of ball milled, and compacted chitin indicates that no chemical change took place during these processes. This guarantees that the intended excipients are chemically stable when exposed to ball milling or compaction. Another objective was to reduce crystallinity to facilitate flow and compression. In this regard, materials are known to undergo plastic deformation with regular particle shape and consequently improved flowability once their amorphous character is increased [30]. Testing the raw chitin showed the necessity to alter the physical state of chitin, namely particle size and crystallinity. In this work, XRPD was used to assure that crystallinity raw chitin was modified, enabling treated chitin to be utilized in pharmaceutical processing.

XRPD results indicated that roller compaction and ball milling techniques affect the crystalline structure of chitin in different ways. A decrease in crystallinity of the semi-crystalline structure of raw chitin was generally the predominant change in the crystal lattice enhanced by both techniques (Figure 2). However, this decrease is more pronounced by ball milling when operated for 36 h. In fact, ball milling converts chitin into a material of a highly amorphous character. In this regard, the decrease in the two main crystalline planes of α-chitin represented by (010) and (020) at 2θ = 9° and 19°, respectively indicates a change in the crystalline nature of chitin. Ioelovich [31] suggests the use of integral intensities (areas) of the X-diffractions, especially for the (020) plane, instead of peak height as an indication of crystalline and amorphous contents for chitin. Based upon his finding, irrespective of which plane is more indicative of any crystallinity change, the summation of the peak areas of the two planes (010) and (020) follows the decreasing order: Raw, compacted, and ball milled. Such crystallinity change caused by roller compaction on chitin is similar to reported results on the effect of roller compaction on α-lactose monohydrate [17]. On the other hand, Alves et al. showed that ball milling of chitosan (de-acetylated chitin) caused a loss of the crystal plane (010); however, there was an increase in the intensity of the peak at the 020 plane [32]. The latter change further contradicts the finding by Ioelovich [31] who—as previously mentioned—states that crystallinity changes are associated with changes in the 020 crystal plane. In order to understand the foregoing contradiction, it is suggested that the dissimilarity with the findings of Alves et al., is more likely to be attributed to the fact that milling—in their work—was carried out for a maximum time of 3 h. The foregoing is too short to represent the extensive duration of the ball milling undertaken in the current work. Therefore, it is the intense stress applied to the powder that imparts a high reduction in its crystallinity. In fact, high stress induction applied to a structurally similar polysaccharide powder, e.g., cellulose, was reported to reduce crystallinity to 54.1% when using simple crushing, and down to 21.7% upon using ball milling [33]. Therefore, for the two techniques employed in this work, it is correct to assert that roller compaction—as a scalable process—can be regarded as a short time ball milling which, in the case of the latter method, is hard to scale-up. 

At the molecular level, it has been reported that destruction of both planes at 2θ = 9° and 19° causes a reduction in the hydrogen bond network which is responsible for imparting structural integrity and flexibility to the chitin chains [34]. Other studies (see Reference [33]) have reported that such a reduction causes a loss in the glycosidic linkages connecting acetylated glucosamine subunits which form the main structural backbone of chitin [33]. On the other hand, the increase in the amorphous character was further evident in the broadening and decrease in band intensities for the IR bands of ball milled chitin (Figure 3C) when the latter is compared with raw chitin (Figure 3A). However, changes in crystallinity for compacted chitin were not detectable using the same technique as the IR bands of roller compacted chitin did not undergo any changes (Figure 3B). 

Moving from the molecular level to the particle behavior of the chitin powder, the first most crucial property to investigate was bulk density with respect to its impact on pharmaceutical processing of powders. In this regard, increasing the bulk density is advantageous in tablet manufacturing, whereby bulky materials enhance powder compression processing, and more specifically, in die filling procedures [35]. The increase in bulk density of chitin, due to ball milling is attributed to the size reduction of the particles, due to high energy impacts between the chitin particles during collision. In comparing the two different stress-inducing techniques, ball milling of chitin is not as good as a powder densification technique compared to roller compaction; the later methodology, imparted a greater increase in the powder bulk density. 

The increase in bulk and tapped densities of chitin, due to humidification is attributed to the low optimal packing caused by strong cohesion -due to interparticle water bridging- between the molecules. Such behavior is generally anticipated when the water content of a material is increased, due to humidification as typically induced in the current case [36]. This further suggests an improvement in particle packing when the powders were subjected to drying for four days. In this regard, dried powder presented the highest recorded bulk and tapped densities in all cases. 

The second most affected factor in dry granulation is particle size distribution. Ball milling is well known as a particle size reducing technique, due to the high energy of impact when the balls collide with chitin [37]. On the other hand, roller compaction is used for powder densification. Consequently, the two techniques are diametric to each other with respect to the changes, increasing or decreasing, they impart to particle size. Although all powders were passed and collected through sieves between 250 and 90 μm, roller compaction imparts high size distribution towards the upper limit of particle size between the aforementioned sieve ranges (Table 3). In contrast, ball milling imparts a high distribution towards the lower limit of particle size. These changes in particle size impart another opposing explanation to the bulk density increase caused by ball milling and roller compaction. In the former, reduction of particle size renders a fixed sample volume to be occupied by small chitin particles rather than large ones. Thus, a higher bulk density is attained upon milling compared to the raw material. On the other hand, despite the increase in particle size in case of roller compaction, the extent of densification is suggested to be high enough to overcome the effect of particle size towards variations in bulk density. It has to be emphasized herein that the aforementioned particle size and bulk density of compacted chitin were only possible after the powder was allowed to be compacted five times up to an applied pressure of 166 Mpa.

The two aforementioned properties; i.e., density and size of the granules, have a direct impact on powder flow, especially when the powders are subjected to humidification. In this regard, the improved flow for raw and ball milled chitin powders subjected to high humidification conditions is suggested to be attributed to the lubrication effect generally induced by water molecules [38,39]. Such a lubrication-flow theory is also aligned with the apparent poor powder flow when such an effect is reduced as the materials undergo drying (Table 4). However, the same theory does not align with the excellent powder flow when compacted chitin was dried. In this case, it is suggested that the effect of bulk density may have overcome the aforementioned concerns related to water content on powder flow. In other words, the significant increase in bulk density noticed for compacted chitin compared to the raw and ball milled chitin has rendered the compacted powders highly flowable. Drying further increases the bulk density value and bulk density difference of the compacted compared to the raw and ball milled chitin powders, and this may justify the excellent flowability of dried compacted chitin. The same justification is valid when compacted chitin was subjected to humidification. In this regard, the sharp decrease in the bulk density of the humidified compacted powder is the main reason behind its poor flowability.

Measuring changes in true density for each stress-inducing technique was tested despite the fact that, theoretically, the true density value of chitin was expected not to be affected by means of pore volume reduction. This is based on the fact that the value is constant for solid matter and excludes any empty space considerations upon measurement. Such a perspective is valid for the material subjected to roller compaction. However, it has been reported that a reduction in the true density of a material can take place when the structure undergoes a polymorphic transformation [40,41]. In this regard, changes in the molecular arrangement of a powder can result in loose packing at the particulate level. Generally, amorphous materials have a lower density than their crystalline counterparts as the atoms of the former are located at further distances from each other than the latter. Therefore, a decrease in crystallinity results in an increase in lattice volume, and thereby, a decrease in density [42]. Thus, the decrease in crystallinity of chitin to a less ordered amorphous structure is suggested to be the main reason behind the decrease in true density of chitin upon ball milling. Water content, on the other hand, lowers the true density of raw chitin, since the measured value is the sum of the true densities of both water and chitin. When water is removed, the measurement will solely include the solid material and without the presence of other materials which disrupt the volume needed to be occupied by chitin for measurement [43]. Based upon the foregoing, the true densities of dried materials, unprocessed and processed, were the highest compared to the humidified ones. 

The last property to be tested, and one which varies with pressure, is a specific surface area. Initially, the high specific surface area of raw chitin is attributed to its highly porous structure [44]. The increase in particle surface area, due to ball milling is typical behavior for a size reducing technique that principally imparts high energy upon collision of the falling balls with powder particles. In contrast, the decrease in particles surface area, due to roller compaction is more likely to be attributed to extensive folding and packing of the particles. Such densification is responsible for reducing the porous structure of raw chitin, and consequently, a decrease in the specific surface area is attained.

Although an increase in the surface area of granules is advantageous in providing new fresh particles surfaces and extra contact points for binding [45], the crushing force of compacted powders of lower surface area was the highest. In contrast, the increase in surface area upon ball milling had no significant effect on crushing force in comparison with roller compaction. Hence, the mechanism for strong binding between the granules is not directly related to the actual surface area of the granules. In fact, it is more likely to be attributed to the mode of deformation when a force is applied. In this regard, plastic deformation of chitin enables extensive folding, thereby providing new surfaces for bridging via new contact points. On the other hand, the presence of water, when the materials were humidified, is a typical action of a granulating medium. The foregoing is known to enforce greater adhesion between wet than dried surfaces [46]. This justifies the increase in crushing force for all the materials when they were humidified.

The hard granules produced by roller compaction showed high resistance to compression force rendering the granules with low maximum volume reduction or ‘*a*’ values. Concurrently, the same hard granules manifested the highest compression force (*P_k_*) needed for (*a*/2) volume reduction as well as minimal particle rearrangement compared to ball milled, and raw chitin. The closer crushing force values of ball milled to raw chitin further rendered similarly closer *a*, *P_K_* and *ab* values. Accordingly, ball milled chitin particles do not provide any added value to the compressibility and compactibility of raw chitin. 

When all the materials were humidified, the compressibility (represented by the ‘*a*’ and to some extent the ‘*ab*’ parameters), underwent an improvement. This is due to the fact that water acts as a plasticizer. The foregoing statement can be justified by the removal of water, as the compressibility of the dried materials decreased. On the other hand, high humidification levels (95% RH) weakened the inter-particulate bonding for all materials as the *P_K_* required for (*a*/2) volume reduction underwent a decrease; thus, resulting in weak humidified granules and for the decrease in tablet hardness attained when the powders were compressed. 

The high compactibility of roller compacted chitin powder was further confirmed using the *P_Y_* parameter from the Heckel model. A lower *P_Y_* value is preferable for plastically deforming materials, as it indicates a higher extent of deformation. The foregoing gives rise to a greater degree of folding, and thus, the appearance of fresh new particle surfaces for bridging with nearby surfaces [47]. Roller compacted powders manifested a high extent of plastic deformation compared to ball milled chitin which, in turn, displayed greater plasticity than the raw chitin. It is suggested that such high plasticity is attributed to the presence of denser chitin particles, due to roller compaction, and thus, new added surfaces are available for deformation. On the other hand, the high plastic deformation presented when the materials were subjected to humidification is, as stated previously, attributed to the plasticizing effect of water. 

The compression force and the volume reduction can be combined together to describe the work of compression manifested by the area under the F-D curve. The fact that the work of compression is the product of force applied (*f*) × the displacement (*d*) of the compressed powder, means that this reflects how easy or hard the granules can be compressed, and/or reflects the extent of plastic/elastic or brittle deformation the particles are undergoing when pressure is applied. Because compressibility is a volume reduction parameter that has a physical displacement implication (*d*), it is suggested that the high *W_c_* values for raw chitin- compared to ball milled- are attributed to its high *a* value. Thus, the impact of *W_c_* was found to be valid at all compression pressures used. Similarly, using the *fxd* correlation to interpret *W_c_* data, high *W_c_* values of dried- compared to humidified- powders are more likely to be related to the increase in *P_k_* values when the powders were dried. In this regard, *P_K_* has a physical implication for *f* values in the F-D data. 

Lastly, in metronidazole preparations comprising drug/compacted-chitin matrix, the highly compactible tablets did not hinder the disintegration time and metronidazole immediate drug release. Such unaffected disintegration is more likely, as previously suggested, attributed to the presence of a larger mass of chitin material in compacted granules. The properties and dissolution of such preparation were even more favorable than tablets made of drug/MCC matrix. 

It is evident that compaction results in a better flow of chitin powder and harder compacts. This may facilitate the utilization of such a powder without any further processing. Such processed powder is less liable to absorb water, as is the case for the ball milled chitin. Keeping the chitin excipient in a dry form allows it to be more appropriate for use in hot and humid climates where the drugs can be more stable when formulated with similar excipients. This behavior is unique when compared with other polymers, such as cellulose, where a balance between crystalline and amorphous states needs to be attained.

## 4. Materials and Methods

### 4.1. Materials

Chitin with an average molecular mass of 300 kDa and a degree of acetylation about 0.96 was obtained from G.T.C. Bio Corporation (Qingdao, China), metronidazole (G.D. Searle and Company, Skokie, IL, USA), microcrystalline cellulose (MCC PH 200^®^, FMC BioPolymer, Philadelphia, PA, USA), pharmaceutical grade talcium (Hubei Aoks Bio-Tech CO., Wuhan, China).

### 4.2. Methods

#### 4.2.1. Sample Preparation Using Roller Compaction

Chitin (3.0 kg) was compacted using a production scale roller compactor (TFC-520 Roller Compactor, capacity of 100 kg/h, Vector Corporation, Freund Group Company, Marion, IA, USA). Repetition of compaction of the same powder was carried out several times until the bulk density of a representative sample exceeded 0.5 g/mL. The foregoing was accomplished after repeating the compaction five times. Every time the chitin sheets were passed through a conical screen mill (Quadro Comil, Ytron-Quadro Ltd, Chesham Bucks, UK) fitted with a 2.4 mm sieve. Compaction parameters for the five stages of compression are summarized in Table 8. Importantly, the milling speed was adjusted to 15, 25, 30, 30 and 30 Hz for stages 1, 2, 3, 4, and 5, respectively. 

#### 4.2.2. Sample Preparation Using Ball Milling

1 kg of chitin was ball milled (Erweka, Langen, Germany) using porcelain balls of diameters ranging from 30 to 50 mm. The material and the balls were inserted in a sealed drum. Milling was carried out for four days, whereby the running time was adjusted to 9 successive hours every day. A sample was taken at the end of each 9 h run. Accordingly, five samples were collected at 0, 9, 18, 27, 36 h of ball milling. 

#### 4.2.3. Preparation of Humidified and Dried Chitin Samples

Three samples from each chitin type (raw, ball milled and compacted) were placed in plastic petri dishes (5 g for each sample) then inserted in desiccators for 30 days. The desiccator was over-saturated with potassium nitrate salt (Acros Organics, New Jersey, NJ, USA) at a relative humidity of 93% at room temperature (25–30 °C); from each sample, known weights were taken for compression analysis.

Another three samples from each chitin type (5 g for each sample comprising either raw, ball milled and compacted) were dried using a Vacucell vacuum drying oven (MMM Medcenter Einrichtungen, Germany). The samples were placed in glass petri dishes then inserted in the oven to dry at a temperature of 95 °C for four days. From each sample, known weights were examined for compression analysis.

#### 4.2.4. Characterization of Powder Properties

##### Scanning Electron Microscopy (SEM)

The morphology of samples was determined using a Quanta-200 3D (ThermoFisher Scientific, Bend, OR, USA) SEM operated at an accelerating voltage of 1200 V. Samples (≈0.5 mg) were mounted on to a 5 × 5 mm silicon wafer affixed via graphite tape to an aluminum stub. The powder was then sputter-coated for 105 s at a beam current of 20 mA/dm^3^ with a 100 Å layer of gold/palladium alloy.

##### X-Ray Powder Diffraction (XRPD)

XRPD test was carried out using an X-ray powder diffractometer (Bruker, Karlsruhe, Germany) in 2-theta range of 2–40° 2θ in reflection mode. The X-ray compartment is a D2 Phaser comprising a copper tube, using Kα X-rays of 300 watts of power at 1.54184 Å wavelength. DIFFRAC.SUITE™ computer software was used to analyze the data obtained.

##### IR Spectrophotometry 

IR spectrophotometry was carried out using Perkin Elmer Spectrum Two UATR FTIR spectrometer, Akron, OH, USA) with a resolution of 4 cm^−1^, data interval of 2 cm^−1^ and a scan speed of 0.2 cm/s operating in the range of 450–4000 cm^−1^. The ATR sample base plate was equipped with a Diamond ZnSe crystal where an infrared background is collected for all FTIR measurements. Samples (2–5 mg) were placed on the ATR crystal, and apressure was applied to compress the sample in order to obtain the spectra. The IR spectra of chitin samples (raw, ball milled, and roller compacted) were examined. 

##### Particle Size Distribution

A laser diffraction Malvern particle size analyzer (Malvern Panalytical Ltd, Malvern, UK) was employed to measure particle size distribution ranging from 0.02 to 2000 microns at room temperature. The instrument is connected to a computer that uses the Mastersizer 2000 (version 5.6) software to display the results.

##### Bulk, Tapped and True Density Measurement and Flow Determination

The bulk density of chitin samples (raw, ball milled and compacted) in g/mL was measured by pouring the powder into a 25 mL volumetric cylinder. The bulk density of all samples was calculated as the ratio of the mass over the volume it occupied. Tapped density measurements were carried out by physical tapping of the cylinder for 100 mechanical taps then dividing the mass over the tapped volume. 

The reduction in the bulk volume of the powders, due to tapping is considered to be an indication of powder flowability, which was evaluated by the Hausner’s ratio (HR). As HR increase in value, the flowability is reduced.

HR is calculated using Equation (1).

HR = ρ_Tapped_/ρ_Bulk_ (1)

The criteria for flow interpretation based on HR is as follows [48]:

Excellent: 1.00 < HR < 1.11; 

Good: 1.12 < HR < 1.18;

Fair: 1.19 < HR < 1.25;

Passable: 1.26 < HR < 1.34;

Poor: 1.35 < HR < 1.45;

Very poor: 1.46 < HR < 1.59.

The true density was determined using a Pycn-020 Gas Pycnometer (Vivid Separation and Filtration, Amman, Jordan). Each sample was weighed then placed in a vial in the second chamber, while air is allowed to pass through the second chamber from the first one. Pressure was recorded in both chambers by a pressure gauge, and the volume of the sample was calculated using the following equation:

*P*_1_ × *V*_1_ = *P*_2_ × (*V*_1_ + *V*_2_ – vs.) (2)

where *P*_1_, *V*_1_ and *P*_2_, *V*_2_ are the pressure and volume in the first and the second chamber, respectively, and vs. is the volume of the sample. The mass of the sample divided by its true volume yields the true density in g/cm^3^. The average of three measurements was carried out for each sample and for each type of density.

##### Water Content Determination

Raw, ball milled, and compacted chitin samples were analyzed for water content using Karl Fischer volumetric titrator (Mettlor Toledo, Hamburg, Germany). Each sample was tested under the following conditions; room temperature (25 °C), humidified for 30 days at 93% relative humidity (RH), dried for four days at 95 °C. Three samples were considered for each analysis. 

##### Specific Surface Area Determination

BET specific surface area was determined by physical adsorption of nitrogen gas using a Nova 2200 multi-speed high gas sorption analyzer (version 6.11, Quantachrome Co., Syosset, NY, USA). Samples were subjected to nitrogen gas for adsorption under isothermal conditions at 77 K. The samples were initially placed in a vacuum oven at 60 °C for 24 h. A reference empty cell (Sartorius, analytic, A120s, Göttingen, Germany) and the sample (~500 mg) were placed in the chambers. The amount of adsorbate gas was measured, then calculations based on a monomolecular layer assumption were applied. BET surface area analysis was calculated from the linear region of the BET plot. 

#### 4.2.5. Powder/Tablet Characterization

Chitin samples (raw, ball milled and compacted) were compressed into tablets using an instrumental single punch bench top tablet press (GTP-1, Gamlen Tablet Press Ltd, Nottingham, UK). Compression was carried out at a punch speed of 60 mm/min by applying five different loads; 100, 200, 300, 400, 500 kg. The samples poured into the die of the GTP had a common weight of 75 ± 1 mg. The diameter and height of the die were 6 and 18.1 mm, respectively. The machine was run by software to display the force-displacement (F-D) curve. Kawakita and Heckel models, Equations (3) and (4), respectively, were utilized to describe the compression analysis of the powders [49,50].

Kawakita analysis describes a linear relationship between the ratio of pressure (MPa) to volume reduction (C) and the pressure. From the slope and intercept of this relationship, constants ‘*a’* and ‘*b’* can be deduced; ‘*a*’ represents the maximum volume reduction that can be attained by the powder. ‘*1/b’* or ‘*P_K_*’ is another parameter that represents the force required to reduce the powder bed volume to half its maximum value.

(3) $$\frac{P}{C}\;=\;\frac{P}{a}\;+\;\frac{1}{ab}\;$$

Heckel analysis describes a relationship between the logarithm of the inverse of compact porosity (ε) and the pressure applied.

(4) $$\ln\frac{1}{\varepsilon }\;=\;kP\;+\;A$$

Porosity is calculated using the following equation:

ε = 1 − ρ_r_ (5)

where ρ_r_ is the relative density of the compact and is calculated using the following equation:

ρ_r_ = ρ_c_/ρ_T_ (6)

where ρ_c_ and ρ_T_ are compact and true densities (kg/m^3^), respectively.

The inverse of the slope of the Heckel equation or ‘1/*K*’ is an important parameter which assigns the type of deformation of materials, whether plastic/elastic or brittle-fracture. This parameter is called the yield pressure and is signified by the symbol ‘*P_Y_*’. 

#### 4.2.6. Application of Compacted Chitin Excipient Using Metronidazole as a Model Drug

It was desired to test the dissolution profile of six tablets (500 mg weight each) comprising the weakly compressible/compactable metronidazole drug (200 mg) and compacted chitin. The matrix will be compared with a reference (6 tablets) made of the same drug and microcrystalline cellulose (MCC). The powder for compression (50 g) was prepared by physically mixing metronidazole (44.4% *w*/*W*) with compacted chitin/or MCC (50% *w*/*w*) and talc (5.6%). The mixtures were compressed using a single punch tablet press (Manesty F3 single stroke tablet press; West Pharma services Ltd, Dorset, UK) at an applied pressure of 35 kN. The fitted die was flat, round and 12 mm in diameter. Prior to dissolution testing, the average crushing force and disintegration time of 10 tablets produced were measured using a crushing force (Pharma Test PTB 311E. Hainburg, Germany) and disintegration (CALEVA, Dorest, UK) testers. The disintegration time of the tablets was determined according to the European Pharmacopoeia Supplement, whereby six tablets were inserted in a water-filled basket-rack apparatus set at 37 °C [46]. 

Dissolution testing was conducted according to USP 32, whereby apparatus II was used (Erweka DT6, Langen, Germany) with paddles rotating at 50 rpm. 900 mL of 0.1 N HCl was used as the dissolution medium. The amount of drug released was analyzed by measuring the absorbance using a UV spectrophotometer (LABINDIA UV/VIS, UV 3000, Maharashtra, India) at a wavelength of 277 nm for metronidazole [51]. 

## 5. Conclusions

Dry granulation via roller compaction has proved to be reliable in the conversion of raw chitin into an excipient suitable for direct compression. The data reported herein illustrates that this can be achieved by multiple compaction of the raw material under high pressure. Analysis of ball milled chitin showed that a decrease in powder crystallinity does not necessarily provide excipients with the required DC specifications. In the same vein, the improvement in raw chitin powder physical properties, i.e., bulk density, flowability, compressibility and compactibility, is attributed to the increase in the amorphous character of raw chitin. The obtained crystal form results in the highly packed arrangement of granules with lower true density and specific surface area than raw and ball milled chitin. On the other hand, such compacted granules were hard and showed resistance to displacement upon compression; however, they manifested high plastic deformation. The foregoing justifies the high crushing strength of tablets produced from compacted chitin. Drying and humidification of granules obtained from ball milling and roller compaction techniques did not provide added value that could serve excipient processing in the context of DC applications. Finally, compacted chitin provided tablets with DC and immediate drug release requirements when formulated with metronidazole (200 mg) as a non-compressible and-non compactible model drug.

## Figures and Tables

**Table 1:** Peak areas of XRPD spectrum of raw chitin, and that subjected to ball milling and roller compaction.

	Area under the Peak	A91	A192	A9/A9R3	A19/A19R4
Chitin					
Raw		512	1472	1	1
Compacted		242	545	0.47	0.37
Ball milled		58	116	0.24	0.21

A9 = Area under the peak at 2θ = 9°; A19 = Area under the peak at 2θ = 19°; A9R = Area under the peak at 2θ = 9° of raw chitin; A19R = Area under the peak at 2θ = 19° of raw chitin.

**Table 2:** Bulk, tapped, and true densities of raw, ball milled, and compacted chitin, before and after humidification or drying.

	Chitin.	Bulk Density			Tapped Density			True Density		
Condition		Raw	Ball Milled	Compacted	Raw	Ball Milled	Compacted	Raw	Ball Milled	Compacted
BHD *		0.19 ± 0.01	0.28 ± 0.01	0.52 ± 0.01	0.29 ± 0.01	0.30 ± 0.01	0.64 ± 0.02	1.35 ± 0.01	1.24 ± 0.02	1.35 ± 0.01
Humidified		0.15 ± 0.02	0.20 ± 0.03	0.32 ± 0.02	0.19 ± 0.01	0.25 ± 0.02	0.52 ± 0.01	1.33 ± 0.02	1.22 ± 0.03	1.37 ± 0.01
Dried		0.33 ± 0.01	0.36 ± 0.01	0.69 ± 0.02	0.48 ± 0.02	0.56 ± 0.01	0.73 ± 0.02	1.37 ± 0.02	1.26 ± 0.01	1.32 ± 0.02

* BHD: before humidification or drying.

**Table 3:** Particle size based on 10%, 50% and 90% distribution of the total sample volume, before and after sieving the powders through a mesh size 250 μm and collected on a 90 μm mesh.

	Particle Size (μm) before Sieving			Particle Size (μm) after Sieving		
**Material**	**d**_**0.1**_	**d**_**0.5**_	**d**_**0.9**_	**d**_**0.1**_	**d**_**0.5**_	**d**_**0.9**_
Raw chitin	107	613	1179	98	178	223
Compacted chitin	156	877	1253	126	199	246
Ball milled chitin	58	384	902	93	121	141

**Table 4:** Hausner ratios of raw, ball milled, and compacted chitin, before and after humidification or drying.

	Chitin	Raw	Ball Milled	Compacted
Condition				
BHD *		1.55 ± 0.046	1.47 ± 0.041	1.23 ± 0.036
Humidified		1.26 ± 0.037	1.27 ± 0.038	1.59 ± 0.047
Dried		1.46 ± 0.043	1.54 ± 0.046	1.06 ± 0.032

* BHD: before humidification or drying.

**Table 5:** Water content of raw chitin, ball milled chitin and roller compacted chitin in different conditions.

Condition	Chitin	Water Content (% *w*/*w*)
Room conditions	Raw	7.350 ± 0.049
	Ball milled	7.150 ± 0.057
	Roller compacted	7. 245 ± 0.014
Humidification at 93% RH at 25 °C	Raw	14.742 ± 0.106
	Ball milled	14.895 ± 0.099
	Roller compacted	14.5947 ± 0.014
Drying at 95 °C	Raw	4.705 ± 0.035
	Ball milled	2.080 ± 0.028
	Roller compacted	3.180 ± 0.014

**Table 6:** The specific surface area of raw chitin, ball milled chitin (36 h), and compacted chitin (stage 5).

Material	BET Surface Area, m^2^/g
Non-compacted chitin	41.5
Chitin/ball milled	49.3
Chitin/compacted	0.84

**Table 7:** Crushing strength, disintegration time, and time for full drug release for metronidazole (200 mg) tablets comprising compacted chitin or MCC PH 200^®^ excipients.

Excipient Used in Metronidazole Preparation	Crushing Strength (N)	Disintegration Time (min)	Complete Dissolution Time (min)
Compacted chitin	110–120	<1	4–5
MCC PH 200^®^	100–105	<12	15–20

**Table 8:** Roller compaction parameters of chitin performed for five successive times.

	Stage 1	Stage 2	Stage 3	Stage 4	Stage 5
Roller type	DPS	DPS	DPS	DPS	DPS
Roller speed. rpm	16 rpm	16 rpm	14 rpm	16 rpm	16 rpm
Screw speed, HZ	4 HZ	8 HZ	8 HZ	8 HZ	8 HZ
Pressure, MPa	83	83	97	140	166
Roll gape, mm	2.8	3.0	3.0	3.0	3.0
